# Everolimus restrains the paracrine pro-osteoclast activity of breast cancer cells

**DOI:** 10.1186/s12885-015-1717-8

**Published:** 2015-10-14

**Authors:** Valeria Simone, Sabino Ciavarella, Oronzo Brunetti, Annalisa Savonarola, Mauro Cives, Marco Tucci, Giuseppina Opinto, Eugenio Maiorano, Franco Silvestris

**Affiliations:** 1 Department of Biomedical Sciences and Human Oncology, University of Bari “A. Moro”, P.zza Giulio Cesare, 11-70124 Bari, Italy; 2 Department of Pathological Anatomy, University of Bari “A. Moro”, Bari, Italy

**Keywords:** BOLERO-2 trial, Breast cancer cells, mTOR, Osteoclastogenesis, Everolimus

## Abstract

**Background:**

Breast cancer (BC) cells secrete soluble factors that accelerate osteoclast (OC) differentiation, leading to the formation of osteolytic bone metastases. In the BOLERO-2 trial, BC patients with bone involvement who received Everolimus had a delayed tumor progression in the skeleton as a result of direct OC suppression through the inhibition of mTOR, in addition to the general suppressor effect on the cancer cells. Here, we explored the effect of Everolimus, as mTOR inhibitor, on the pro-OC paracrine activity of BC cells.

**Methods:**

Both MDA-MB-231 and MCF-7 BC cell lines were incubated with sub-lethal amounts of Everolimus, and their conditioned supernatants were assessed for their capacity to differentiate OCs from PBMC from healthy donors, as well as to interfere with their bone resorbing activity shown on calcium phosphate slices. We also measured the mRNA levels of major pro-OC factors in Everolimus-treated BC cells and their secreted levels by ELISA, and evaluated by immunoblotting the phosphorylation of transcription factors enrolled by pathways cooperating with the mTOR inhibition. Finally, the *in vivo* pro-OC activity of these cells was assessed in SCID mice after intra-tibial injections.

**Results:**

We found that Everolimus significantly inhibited the differentiation of OCs and their *in vitro* bone-resorbing activity, and also found decreases of both mRNA and secreted pro-OC factors such as M-CSF, IL-6, and IL-1β, whose lower ELISA levels paralleled the defective phosphorylation of NFkB pathway effectors. Moreover, when intra-tibially injected in SCID mice, Everolimus-treated BC cells produced smaller bone metastases than the untreated cells.

**Conclusions:**

mTOR inhibition in BC cells leads to a suppression of their paracrine pro-OC activity by interfering with the NFkB pathway; this effect may also account for the delayed progression of bone metastatic disease observed in the BOLERO-2 trial.

**Electronic supplementary material:**

The online version of this article (doi:10.1186/s12885-015-1717-8) contains supplementary material, which is available to authorized users.

## Background

The development of skeletal metastases, leading to hypercalcemia, fractures, and vertebral collapse with spinal compression and intractable pain, is a major cause of morbidity in patients with advanced breast cancer (BC) [1]. Their formation is primarily induced by a marrow environment fostering tumor growth, thanks to the functional cross-talk between BC cells and resident cells such as hematopoietic progenitors, stromal, endothelial and other marrow components [2]. Although a direct bone resorption activity by BC cells was postulated [3], it is currently believed that osteolytic lesions are produced by marrow osteoclasts (OC), whose differentiation from myeloid precursors is accelerated by the tumor cells [4]. BC cells secrete PTHrP (parathormone-related protein) [5], in addition to other soluble factors that are normally secreted by stromal and other marrow cells to regulate the OC maturation, and have M-CSF (macrophage-colony stimulating factor), IL (interleukin)-1β, IL-8, IL-6 and TNF-α (tumor necrosis factor) functions [6–8].

Specific signatures of molecular pathways in BC cells have been associated with a highly malignant phenotype [9] and worse clinical outcome [10]. For instance, hyperactivity of the phosphoinositide 3-kinase (PI3K)/Protein Kinase B (Akt)/mammalian target of Rapamycin (mTOR) pathway, is a molecular hallmark of accelerated proliferation and tumor progression in BC cells, since intrinsic molecular aberrations recur in approximately 40 % of patients with metastatic skeleton disease [11]. The constitutive phosphorylation of Akt, as well as mutations of the catalytic subunit of PI3K *(PIK3CA)*, result in persistent activation of mTOR as the downstream serine/threonine kinase that primarily regulates proliferation, survival and apoptosis in BC cells [12]. Further work also suggests that this abnormal mTOR activity induces a peculiar osteotropic phenotype of BC cells, probably involved in skeleton colonization [13], that could ultimately be modulated by inhibiting this pathway, as demonstrated in other cancers [14].

Based on the results of the BOLERO-2 trial, the mTOR inhibitor Everolimus (RAD001) has been approved for use in postmenopausal women with hormone-receptor positive (HR^+^)/Her-2^−^ advanced BC, refractory to non-steroidal aromatase inhibitors [15]. In this study, a beneficial effect on bone health was documented in bone-metastatic patients receiving the drug, skeletal tumor progression being significantly delayed as compared to in the control group patients [16]. The bone resorption markers at 6 and 12 weeks revealed a significant decrease in patients treated with Everolimus [16], and this beneficial effect on bone health was attributed to the reduced OC function associated to the general anti-tumor activity induced by Everolimus on BC cells, as well as to a direct mTOR inhibition in BC-stimulated OCs, since this pathway drives their functional maturation [17]. However, this result in the BOLERO-2 trial may also be explained as an effect of suppressed pro-OC paracrine activity of BC cells in response to the mTOR inhibition, besides its modulatory role on hyperactive OCs. Another trial, the RADAR study, suggested that Everolimus as a single agent was beneficial in patients showing only bone involvement [18]. These effects have been further emphasized in a recent subanalysis of the BOLERO-2 trial confirming the beneficial effect of Everolimus in the sub-population of BC patients with bone metastases, as well as in other subgroups [19].

However, in OC progenitors, as in BC cells, the secretion of pro-OC factors is independent of the mTOR pathway since the nuclear factor kappa B (NFkB) pathway is primarily involved through transcriptional factors regulating their release and activity [20–24]. Therefore, assuming that mTOR inhibition restrains the whole tumor activity in BC cells, as described in BOLERO-2, it is conceivable that both the mTOR and NFkB pathways may be molecularly interconnected and that their interaction may ultimately bring about the inhibitory effect on the secretion of pro-OC factors [25].

Here, we investigated the potential of Everolimus to regulate the pro-OC paracrine activity of BC cells, and provide evidence that this mTOR inhibitor restrains the bone-destroying potential of BC cells both *in vitro* and *in vivo*, through a synergic negative efficacy on both mTOR and NFkB. Thus, the NFkB-mediated suppression of the pro-OC paracrine activity by BC cells in patients enrolled in the BOLERO-2 trial may account for the beneficial bone effect attributed to Everolimus.

## Methods

### Compound

Everolimus [40-O-(2-hydroxyethyl)-rapamycin]) as pure extract powder was provided by Novartis Pharma and prepared in dymethylsulfoxide, diluted at 100 mg/ml, for in vitro use.

### BC cell lines and survival assay

MDA-MB-231 and MCF-7 BC cells (ATCC, Rockville, MD, USA) were cultured in RPMI 1640 supplemented with 10 % FCS (Sigma Aldrich, Milan Italy), 100 U/ml penicillin/streptomycin and 2 mM L-glutamine (PAA, Pasching, Austria) in 5 % CO_2_-incubator. Both cell lines were incubated for 48 h with increasing concentrations (10^−1^ to 10^4^ nM) of Everolimus and their viability was assessed by methylene blue. Briefly, after fixation with glutharaldehyde, the cells were incubated for 10 min with methylene blue [0.05 % (*w/v*) in water], then treated with HCl [3 % (*v/v*)] and read at 650 nM of absorbance in a microtiter reader (BIO-Rad, Milan, Italy). The inhibitory concentration 20 (IC_20_), defined as the amount of Everolimus able to reduce cell growth by 20 %, was calculated according to BioDataFit 1.02 software by interpolation of the dose-response curves. IC20 was arbitrarily used as the sub-lethal dose and each experiment was performed in triplicate.

### BC cell conditioned media (CM)

After 48 hr-treatment with control DMSO or Everolimus at IC_20_, BC cells (1 × 10^5^/well) were seeded in 6-well plates for 24 h. Then, supernatants were recovered, filtered, frozen at −20 ° C until use for *in vitro* and *in vivo* experiments (see below).

### OC differentiation and activity

Human OCs were obtained from the peripheral blood of healthy blood donors, after obtaining written informed consent, and approval by the Ethics Committee of the University of Bari. OCs were generated in vitro after 16-day incubation of PBMCs with RANKL (50 ng/ml) and M-CSF (25 ng/ml) (Isokine, Iceland), as previously reported [26]. At day 8, PBMCs were supplemented with 20 % of CM from DMSO- or Everolimus-treated cells and after a further 8 days of incubation, both the morphology and function of OCs were assessed.

We arbitrarily considered as OC-like cells, polykaryons with at least three nuclei, that were counted in ten microscopic fields at 30× magnification after hematoxylin-eosin staining (Vector Labs, Sigma) and compared with tartrate-resistant acid phosphatase positive (TRAcP^+^) cells in parallel preparations using naphthol AS-BI 0.12 mg/ml, 6.76 mM tartrate, and 0.14 mg/ml Fast Garnet GBC (Sigma-Aldrich).

Functional OC activity was measured on experimental bone substrate. Briefly, pre-OCs obtained after 8 days of culture in the presence of RANKL and M-CSF were incubated for a further 8 days with and without CM on calcium phosphate discs (BioCoat Osteologic Discs; BD Biosciences). Then, the cells were removed by 5 % sodium hypochlorite and the substrates were stained by the Von Kossa method to reveal erosive pits. We also quantified both the number of pits and the percentage of the resorbed area by a dedicated software (Olympus) under light microscopy.

### RT-PCR

After 48 hr-treatment with control DMSO or Everolimus at IC_20_, both the MDA-MB-231 and MCF-7 cell lines were measured for mRNA levels of *M-CSF*, *RANKL*, *TNF-α*, *IL1-β*, *IL6*, *MMP* (metalloproteinase)-*9*, *MMP-13*, *MCP* (monocyte chemoattractant protein)-1, *MIP* (macrophage inflammatory protein)-*1α*, as the major pro-OC factors [27–32]. Also, the OC-like phenotype of BC cells was assessed on the levels of *TRAcP*, *cathepsin K* (*Cat K*) and *c-fms*. RNAs were extracted by the RNeasy kit (Qiagen, Chatsworth, CA) and the relative concentrations calculated by a Bio Photometer (Eppendorf) at 260 nm. After evaluating RNA integrity by electrophoresis, cDNA was obtained by reverse transcription of 1 μg of total RNA using a commercial kit (Applied Biosystems, Milan) in a Mastercycler Personal (Eppendorf). Levels of the above genes were assessed by specific primers (Additional file 1: Table S1) designed on different exons to avoid amplification of contaminating genomic DNA. β-actin was selected as target gene for the RT-PCR using Sybr Green (Maxima® SYBR Green/ROX qPCR Master Mix, Fermentas), in the ABI Prism 7300 Sequence Detector (Applied Biosystems). Following an initial incubation at 50 °C for 2 min, samples were denatured at 95 °C for 10 min, then by 40 cycles at 95 °C for 15 s, and 1 min at 60 °C. All tests were completed in triplicate and gene expression levels were obtained as 2^-ΔΔCt^ [33].

### Measurement of pro-OC cytokines released by BC cells

M-CSF, IL1-β, IL-6, TNF-α and MIP-1α were measured in CM from each cell preparation by the appropriate ELISA (R&D systems, Minneapolis, MN), as reported [34].

### Immunoblotting assessment of the mTOR and NFkB pathways in BC cells

Both DMSO- and Everolimus-treated MDA-MB-231 and MCF-7 cells were investigated for activation of the mTOR and NFkB pathways, as well as for the production of NFkB-dependent pro-OC mediators. After 48 hr-incubation, cells were harvested and lysed in mRIPA lysis buffer (10 mM Tris–HCl, 150 mM NaCl, 2 mM EDTA, 1 % NP40, 0.1 % 0.1 % SDS, 1 % sodium deoxycholate) and 20 μg of total protein lysate were resolved on 10 % SDS-PAGE and subjected to Western-blotting [35] using MoAbs to Akt, phosphorylated (p)-mTOR, p-p70S6K (Millipore, Billerica, MA, USA), IKKα, p-IKKα, p65, p-p65, as well as M-CSF, IL-1β, IL-6 (Cell Signaling Technology, Danvers), TNF-α and MIP-1α (Abcam, Cambridge). β-actin was used as intra-assay marker for both quality and quantity loading of sample lysates.

### Generation and detection of BC bone metastases in vivo

To investigate the capability of BC cells to generate *in vivo* bone metastases and the effect of the 48 hr-treatment with sub-lethal doses of Everolimus, we utilized MDA-MB-231 as predominant bone metastasizing BC cell model [36] in 8-week old NOD.CB17-Prkdc^scid^/J mice (Charles River, Milan, I). All experiments were performed in accordance with the Italian Guidelines for the use of laboratory animals, following the European Union Directive for the protection of experimental animals (2010/63/EU), after receiving approval from the Animal Experimentation Ethics Committee (CESA) of University of Bari “Aldo Moro”. Animals were maintained under standard environmental conditions and provided with feed and water ad libitum. Considering the animal ethical issues, all animals were kept under best hygienic conditions and were daily inspected for signs of pain or discomfort. Briefly, eight mice were anesthetized by Isofluorane, and 1 × 10^5^cells/20 μl of Everolimus-treated and untreated MDA-MB-231 were inoculated into the left and the right tibial cavity, respectively, of the flexed knees of each animal. After 4 weeks, the animals were euthanized by carbon dioxide and X-Rays were taken at 20 kV and 25 mAs for 5 s using a mammographic device (Model Flat E; Metaltronica, Rome). Films were then comparatively inspected for structural deformities and the size of visible tibial lesions was measured by ImageJ software, version 1.45 (National Institutes of Health, Bethesda, MD). The extent of osteolytic areas, as mm^2^ of bone devastation, were compared in each mouse between the right and left tibias.

### Bone immunohistochemistry

The tibias were excised, fixed and decalcified in EDTA for paraffin-embedding; 4 μm-thick sections were stained with hematoxylin-eosin while parallel sections were prepared for TRAcP staining (Aviva Systems Biology, San Diego) using specific reagents and avidin-biotin (Vector Labs, Burlingame) to reveal TRAcP^+^ cells [37].

### Statistical analysis

We employed GraphPad Prism 6.1 software (Macintosh, La Jolla, CA) and differences were calculated by Student’s *t* test. *P* < 0.05 was considered statistically significant.

## Results

### Everolimus sub-lethal dose

We firstly assessed the cytotoxic activity of Everolimus on BC cells (Additional file 1: Figure S1). The IC_20_ concentration of Everolimus was 10 nM for MDA-MB-231 cells and 0.5 nM for MCF-7 cells; these were used as sub-lethal doses.

### BC-induced osteoclastogenesis is restrained by Everolimus

**Fig. 1:**
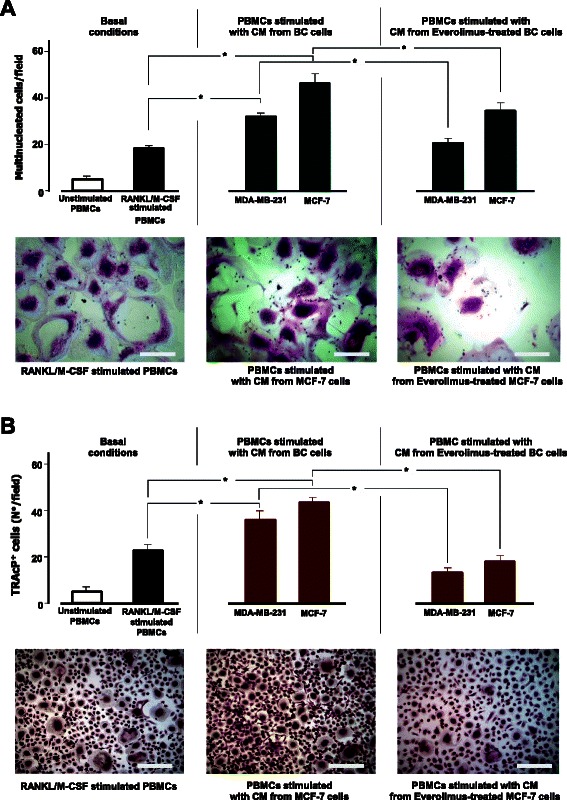
Osteoclastogenic potential of BC cells and effects of Everolimus. PBMCs from healthy donors were differentiated to OCs using osteoclastogenic factors (*left*) and conditioned medium (CM) from MDA-MB-231 and MCF-7 BC cell lines, before (*middle*) and after (*right*) treatment with Everolimus. The pro-OC potential was measured as numbers of both large multinucleated OC-like cells stained by hematoxylin/eosin (**a**) and multinucleated TRAcP^+^ cells (**b**). CM from BC cells produced significant increases (*p < 0.05) of OC formation, although the greatest effect was with MCF-7 cells (*middle*). Both constitutive and BC-mediated osteoclastogenesis were abrogated when using CM from BC cells pretreated with a sub-lethal dose of Everolimus. The pattern of increased/reduced OC formation was similarly evident after different staining. **a** bottom: Polykaryons from PBMCs cultured with RANKL/M-CSF (*left*), with CM from untreated (*middle*) and Everolimus-treated (right) MCF-7 cells (scale bar: 30 μm; 30X magnification). **b** bottom: images of TRAcP^+^ OC-like cells (scale bar: 35 μm; 20x magnification)

Figure 1a shows the pro-OC potential of CM from both BC cell lines, as untreated and Everolimus-treated cells. CM from both untreated cell lines produced higher numbers of polykaryons (MDA-MB-231: 32.5 ± 1.5; MCF-7: 45 ± 6 cells/field) compared to control PBMCs (16 ± 1 cells/field; *p* < 0.05), whereas CM from Everolimus-treated MDA-MB-231 and MCF-7 significantly reduced the polykaryon generation, to 35 % (21 ± 2.6 cells/field) and 23 % (35 ± 4 cells/field), respectively (*p* < 0.05). We further investigated the TRAcP expression in polykaryons (Fig. 1b). Again, the number of TRAcP^+^ cells was enhanced by CM from both BC cell lines (MDA-MB-231: 35 ± 2.5; MCF-7: 43 ± 2 cells/field) as compared to control PBMC preparations (22 ± 2.5 cells/field; *p* < 0.05), while this effect was neutralized by CM from both Everolimus-treated cell lines (*p* < 0.05). The bottom images of Fig. 1b illustrate basal TRAcP^+^ cell formation (left), after MCF-7 CM (middle), and CM from Everolimus-treated MCF-7 cells (right).

### Everolimus restrains the BC-mediated OC hyperactivity

The restraining effect of Everolimus on the pro-OC activity of BC cells was investigated at both RNA and functional levels.

**Fig. 2:**
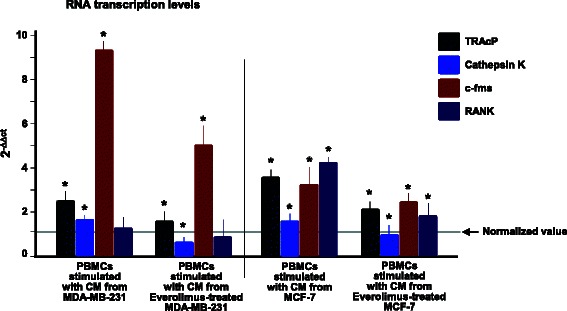
Effect of BC cells on the transcription of OC genes in PBMCs and inhibition by Everolimus. RNA levels of *TRAcP, Cat-K, c-fms* and *RANK* in PBMCs after incubation with CM from BC cells, before and after treatment with Everolimus. Transcripts were variably increased in both cell lines, particularly *c-fms* by MDA-MB-231, and significantly down-regulated by Everolimus. RNA levels were normalized to control OCs and to β-actin levels as house-keeping gene, and expressed as 2^-ΔΔCT^

**Fig. 3:**
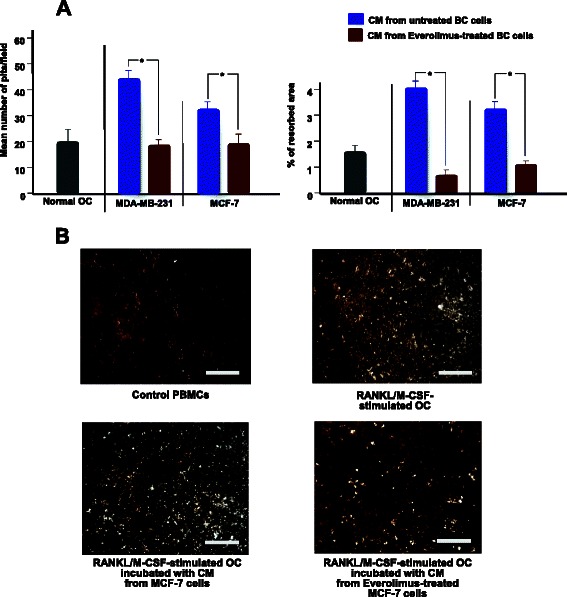
Measurement of bone resorbing activity of OCs stimulated by BC cells. The OCs capacity to produce erosive lacunae on calcium phosphate was measured after stimulation with CM from both untreated and Everolimus-treated BC cells. **a** Significant increases of the number of erosion pits (*above*) and percentage of resorbed area (*below*), were observed after incubating OCs with CM from both BC cells, whereas this effect was significantly suppressed by Everolimus (*p < 0.05). Results are means of three experiments. **b** Variation of erosive pit formation by von Kossa-staining of calcium phosphate slices seeded with OCs compared to culture conditions. CM from Everolimus-treated MCF-7 cells inhibited the bone resorbing activity (*bottom, right*) compared to the constitutive activity (*above, right*) and to untreated cells CM (*bottom, left*) (scale bar: 25 μm; 40x magnification)

As shown in Fig. 2, quantitative RT-PCR revealed that OCs had significantly increased mRNA levels of *TRAcP*, *Cat-K* and *c-fms* after incubation with CM from both the MDA-MB-231 and MCF-7 cell lines (*p* < 0.05). No variation was observed for RANK mRNA after adding MDA-MB-231 CM, whereas treatment with CM from Everolimus-treated BC cells resulted in a suppression of *TRAcP*, *Cat-K* and *c-fms* (*p* < 0.05). Functional tests supported these results. OCs showed a dramatically reinforced bone resorbing activity on calcium-phosphate slices after adding CM from both BC cell lines and, contrarily, a significantly inhibited activity when using CM from Everolimus-treated cells (Fig. 3). In particular, Fig. 3a (above) shows that OCs stimulated with CM produced higher numbers of erosion pits (MB-231: 45 ± 2.5; MCF-7: 32 ± 3) than control OCs (20 ± 3.1), with increased total resorbed areas, equal to 3.8 and 3.1 %, respectively, with CM from untreated MDA-MB-231 and MCF7 cells, compared to 1.5 % of the control OCs, as shown in Fig. 3a (below). By contrast, when using CM from Everolimus-treated BC cells, both erosion pits and resorbed areas were significantly reduced (*p* < 0.05 in all instances). Figure 3b depicts representative images of erosion pits on calcium-phosphate discs. These results emphasized the ability of Everolimus to restrain the BC-mediated hyperactivity of OCs, at both molecular and functional levels.

### Everolimus restrains the pro-OC paracrine activity of BC cells

**Fig. 4:**
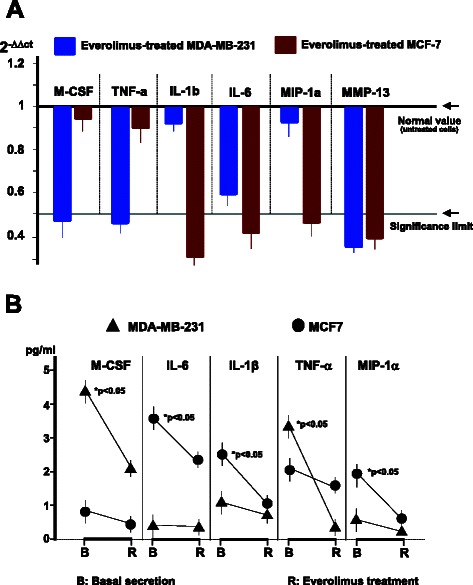
Transcriptional and secretory profiles of pro-OC factors in Everolimus-treated BC cells. RNA levels of *M-CSF, TNFα, IL-β, IL-6, MIP-1α* and *MMP-13* were variably suppressed in both MDA-MB-231 and MCF-7 cells after treatment with Everolimus. **a** RT-PCR showed a significant reduction of *M-CSF, TNFα,* and *MMP-13* RNAs in MDA-MB-231, with a concurrent diminution of *IL-6. IL-1β, IL-6, MIP-1α* and *MMP13* were also significantly inhibited in MCF-7 cells as compared to normalized values from control untreated BC cells. Data are mean values from three experiments. **b** ELISA measurement of major NFkB-dependent osteoclastogenic factors, namely M-CSF, IL-6, IL-1β, TNF-α and MIP-1α, released by cultured MDA-MB-231 and MCF-7 cells before or after sub-lethal doses of Everolimus. In most instances, Everolimus suppressed the secretion of these factors (mean values from three experiments), showing a similar pattern to that of RNA inhibition

MDA-MB-231 and MCF-7 were tested by RT-PCR for *M-CSF, RANKL, TNF-α, IL-1β, IL-6, MMP-9, MMP-13, MCP-1* and *MIP-1α*, before and after Everolimus treatment. Levels of mRNAs were normalized to 1.0 as 2^-ΔΔct^ basal value and with the exception of *RANKL, MMP-9* and *MCP-1α,* that were almost undetectable (data not shown), both cell lines showed a variably suppressed RNA transcription of the other genes after Everolimus treatment. Results are illustrated in Fig. 4a. MDA-MB-231 cells underwent significant reductions (*p* < 0.02) of *M-CSF, TNF-α*, and *MMP-13* RNA levels whereas *IL-6* was unaffected (*p* > 0.05), like *IL-1β* and *MIP-1α*. In parallel, MCF-7 cells were suppressed by Everolimus in *IL-1β, IL-6, MIP-1α* and *MMP-13* (*p* < 0.02 in all instances), unlike *M-CSF* and *TNFα.*

To verify whether the suppressed transcription of RNAs was paralleled by a concurrent reduction in the secretion of related proteins, we measured M-CSF, TNFα, IL-1β, IL-6, MIP-1α, and MMP-13 amounts in CM by ELISA. Figure 4b depicts the results, showing an apparent correlation of RNA amounts with lower secreted levels of those cytokines. MDA-MB-231 cells constitutively secreted consistent amounts of both M-CSF and TNF-α, whereas MCF-7 cells produced higher levels of IL-1*β*, IL-6 and MIP-1α than MDA-MB-231. However, Everolimus significantly inhibited this paracrine activity in both cell lines (*p* < 0.05 in all instances).

### mTOR inhibition by Everolimus disables NFkB signaling in BC cells

**Fig. 5:**
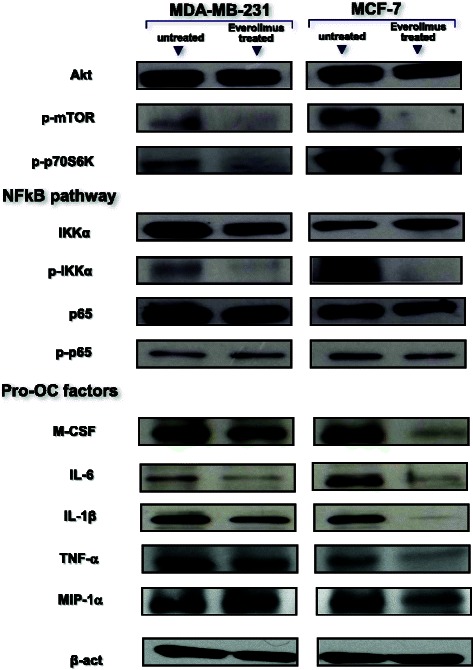
mTOR inhibition down-regulates the NFkB pathway in BC cells. Intracellular content of mTOR, NFkB-related molecules and pro-OC effectors in BC cells before and after Everolimus treatment (measured by Western-blot). Protein lysates revealed a high native content, although at higher OD values in MCF-7 cells, as in, Additioinal file 1: Table S2, of phosphorylated mTOR and p70S6K components of the mTOR pathway, and concurrently, p-IKKα and p-p65 factors belonging to the NFkB signaling pathway. In both cell lines, Everolimus strongly suppressed the constitutive hyperphosphorylation of IKKα and p65, as well as of p-mTOR and p-P70S6K. This supports the potential of Everolimus to inhibit the NFkB pathway, since the intracellular expression of M-CSF, TNF-α, IL-6, IL-1β and MIP-1α, as major NFkB-dependent pro-OC factors, was suppressed as well as p-IKKα and p-p65. β-actin was the loading control

Since the NFkB pathway regulates the transcription of *M-CSF, IL-1β, IL-6, TNFα* and *MIP-1α* for the relative protein secretion by BC cells, we investigated the interference exerted by Everolimus on mTOR and NFkB signaling. By Western-blot, we measured the amounts of major factors of both pathways, such as Akt, phosphorylated (p) mTOR, p-p70S6K, IKKα, p-IKKα, p65, and p-p65, in both BC cell lines before and after Everolimus treatment. Figure 5 (up) depicts the constitutively increased expression of p-mTOR and its downstream effector p-p70S6K in both cell lines, although at higher levels in MCF-7 (OD values are in, Additional file 1: Table S2). As expected, Everolimus decreased p-mTOR and p-p70S6K levels in both cell lines, particularly in MCF-7 cells, whereas no change in Akt expression was observed, confirming the ability of Everolimus to selectively target the m-TOR molecule and hence inhibit the m-TOR-dependent p70S6K factor.

The inhibition of the interaction between the mTOR and NFkB pathways exerted by Everolimus is shown in Fig. 5 (middle) on both cell lines. IKKα and p65 exhibited variably inhibited phosphorylated forms after Everolimus. The effect was more pronounced for p-IKKα in MCF-7 cells, due to the higher constitutive expression of this factor. Similarly, M-CSF, IL-6, IL-1β, TNFα, and MIP-1α were measured as NFkB-dependent factors. Figure 5 (below) shows their lowered intracellular content after Everolimus, especially in MCF-7 cells, supporting the hypothesized negative regulation of NFkB signaling by m-TOR inhibition.

### Everolimus-treated MDA-MB-231 cells reveal a restrained osteolytic activity in SCID mice

**Fig. 6:**
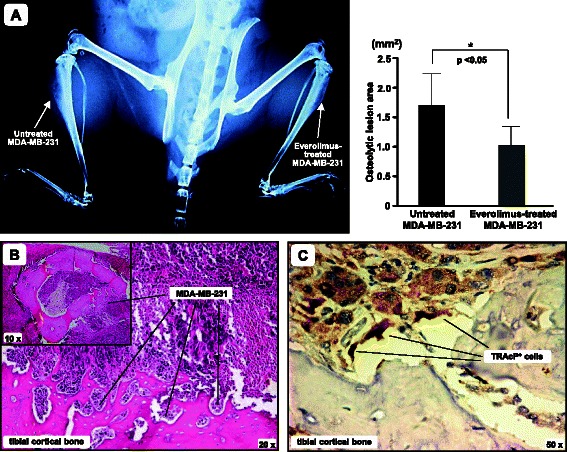
Everolimus restrains the bone-metastatic potential of BC cells in vivo. Everolimus-treated and untreated MDA-MB-231 cells were intra-tibially inoculated in 8-week old SCID mice. **a** X-Ray image of a representative SCID mouse after 4 weeks, showing blown cortical bone of the right tibia and a smaller bone lesion produced by Everolimus-treated MDA-MB-231 cells in the left tibia. The graph shows levels of tibial erosion, measured by ImageJ software. Data are expressed as mean ± SE of the 2D size of metastatic lesions (untreated MDA-MB-231: 1.7 ± 0.56 mm^2^; Everolimus-treated MDA-MB-231: 0.9 ± 0.31 mm^2^; *p < 0.05). **b** Representative image of tumor infiltration in an excised tibia (H/E staining). The small box includes a horizontal section of the right tibia, showing that the cortical bone is largely infiltrated by MDA-MB-231 cells, as revealed at higher magnitude. **c** Detection of TRAcP^+^ cells at the tumor/bone interface of the same tibia

Everolimus-treated and untreated MDA-MB-231 cells were inoculated into the left and right tibia, respectively, of each 8-week old SCID mouse. After 4 weeks, X-Rays were taken to measure the extension of osteolytic areas. We observed smaller osteolytic areas in the tibias engrafted with treated MDA-MB-231 cells compared to the contralateral tibias, that uniformly showed variably enlarged proximal epiphyses due to the growth of metastatic lesions. Figure 6a (left) illustrates a representative X-Ray of a SCID mouse. As can be seen, the right tibia injected with untreated MDA-MB-231 produced a larger osteolytic lesion with blown cortical bone, compared to the smaller lesion produced by Everolimus-treated cells in the left tibia. Figure 6a (right) shows the average size of these osteolytic areas measured on X-Ray films of all mice, supporting the evidence of significantly larger lesions in the right tibias, injected with untreated MDA-MB231 cells (mean value: 1.7 ± 0.56 mm^2^) compared to the left tibias, injected with Everolimus-treated cells (0.9 ± 0.31 mm^2^) (*p* < 0.03). Finally, Fig. 6b and c shows the histology pattern of tumor engraftment by MDA-MB231 cells of the right tibia of a SCID mouse, displaying devastation of the bone structure, massive infiltration of the spongy bone, and a number of TRAcP^+^ cells, so functional OCs, in close contact with the tumor cells and juxtaposed to the cortical bone.

## Discussion

Endocrine therapy is the standard care for patients with advanced HR^+^ BC. However, after an initial response, most patients develop endocrine resistance. The underlying molecular mechanism is PI3K/Akt/mTOR hyperactivity, that drives the phosphorylation of the estrogen receptor (ER) by p70S6K, leading to a ligand-independent ER transcriptional activity. The involvement of mTOR in the cell growth of endocrine resistant BC cells is also supported by the convergence of signaling from different growth factor receptor pathways, such as the epidermal growth factor receptor (EGFR), insulin growth factor receptor (IGF-1R) and insulin receptor (InsR), toward PI3K/Akt/mTOR [38]. Moreover, besides the expected improvement of progression-free survival obtained by mTOR inhibition in Everolimus-treated patients with endocrine resistant BC, results from the BOLERO-2 trial suggest that this drug significantly delays skeletal disease progression, exerting a definite bone-sparing effect in metastatic BC patients [16].

Skeletal metastases occur in BC as an effect of the osteotropism of this tumor, that primes OC hyperactivity to generate typical osteolytic lesions. Although BC cells themselves are suspected to degrade the bone matrix [3], their paracrine pro-OC activity is a major pathogenic event driving excessive OC differentiation and hyperactivation, resulting in the formation of bone metastases [39, 40]. Therefore, we investigated whether Everolimus acts as a negative regulator of the paracrine pro-OC activity of BC cells.

The beneficial effect of Everolimus on bone health in the BOLERO-2 study has also been interpreted as a result of the inhibition of mTOR activity in OCs [16, 17]. It has been demonstrated in transgenic mice, bearing human-*TNFα* to resemble a rheumatoid arthritis model, that mTOR signaling drives OC function, and that inhibition of this pathway can mitigate bone erosion [41]. In these animals, mTOR inhibition by Everolimus or Sirolimus resulted in a significantly decreased structural damage in joints.

We used the BC cell lines MDA-MB-231 and MCF-7, both capable of producing osteolytic lesions in animals [36, 42]. In line with previous data [43], both cell lines secreted variable amounts of pro-OC factors and their CM accelerated the differentiation of PBMCs to TRAcP^+^ polykaryons, namely OCs showing bone-resorption activity *in vitro*, with a concurrent RNA increase of pivotal OC-related genes, such as *RANK* and *c-fms* as phenotype markers, and *cathepsin-K* and *TRAcP* as functional markers.

Everolimus treatment of BC cells inhibited the release of pro-OC factors. Using their CM, we observed a decline in the formation of TRAcP^+^ polykaryons, together with a lower transcription of major OC factors and decreased bone resorbing ability. However, although these effects were similarly detectable in both cell lines, a higher inhibitory effect was recorded when PBMCs were stimulated with CM from Everolimus-treated MCF-7 cells. We interpreted this result as dependent on the higher sensitivity to Everolimus treatment of MCF-7 cells, as an effect of the intrinsic *PIK3CA* deregulation leading to a constitutive mTOR hyperactivity [44]. On the other hand, in both cell lines the RNA of *M-CSF, TNFα, IL-1β, IL-6, MIP-1α* and *MMP-13,* as major pro-OC factors, was variably lowered by Everolimus: the basal transcriptional profile implying a well-defined inherited pro-OC potential was negatively regulated by the mTOR inhibitor. However, transcriptional levels of other genes such as *RANK-L, MCP-1* and *MMP-9* remained undetectable, as reported by other investigators [45].

A major result in our work was the inhibition of M-CSF, IL-6, IL-1β, TNFα and MIP-1α induced by mTOR inhibition. Constitutive phosphorylation of p70S6K, 4EBP1 and eIF4E, as downstream mTOR effectors [46], has been described to contribute to cell growth, angiogenesis and metastasis in several tumors. mTOR inhibition restrains these effects without having any apparent impact on M-CSF, IL-6, and IL-1β secretion, since these factors are regulated by NFkB [20–24], whose activation is apparently independent of the mTOR pathway. This point is disputed, however, since previous works reported that a potential interaction between mTOR and IKKα triggers NFkB in tumor cells and constitutive activation of the PI3K/Akt/mTOR pathway [25, 47]. We thus investigated whether NFkB signaling is affected by mTOR. After treatment of both cell lines with Everolimus, the secretion of M-CSF, TNFα, IL-1β, IL-6 and MIP-1α in culture was significantly reduced, in parallel with lower RNA values. These data, together with the increased phosphorylated levels of pIKKα and p-p65 at immunoblotting, support a synergic down-regulation of both mTOR and NFkB. We found, indeed, that higher levels of Akt, p-mTOR and p-p70S6K in MCF-7 cells were correlated with similarly increased levels of p-IKKα and p-p65 along the NFkB pathway. The inhibition of NFkB phosphorylated effectors after treatment with Everolimus definitely suggests a functional connection between the mTOR and NFkB pathways.

**Fig. 7:**
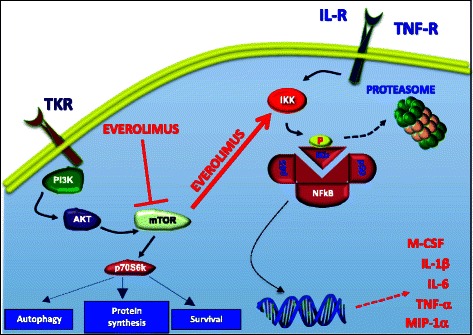
Potential inhibitory interactions between the mTOR and NFkB pathways activated by Everolimus. It is suggestive that Everolimus disables IKK by p-mTOR, thus restraining the phosphorylation of IKb. This event prevents the migration of p65, as NFkB component, to the nucleus where it normally acts as transcription factor for *M-CSF, TNF-α, IL-6, IL-1β* and *MIP-1α.* This ultimately leads to an inhibited secretion of the pro-OC soluble mediators coded by these genes

It has been suggested that the functional disablement of NFkB following proteasome inhibition may affect intracellular signaling propagated through the mTOR pathway [48]. In our study, however, we provide inverse evidence that, at least in BC cells, both p-IKKα and p-p65, as NFkB components, can be disabled by inhibiting mTOR. Although it has been reported that both MDA-MB-231 and MCF7 may acquire resistance to everolimus [49], in our hands, the minor intracellular content of pro-OC factors, namely M-CSF, IL-6, IL-1β, TNFα and MIP-1α, induced by NFkB in Everolimus-treated BC cells emphasizes the interactive negative regulation between these pathways. A model of interactive molecular cross-talk between mTOR and NFkB signaling is proposed in Fig. 7.

To substantiate our hypothesis, we explored the effect of mTOR inhibition on BC paracrine pro-OC activity in a xenograft model of bone metastatic disease. In each experiment, Everolimus-treated and untreated MDA-MB-231 cells were intra-tibially injected into the left and right tibial marrow cavity, respectively, of SCID mice. This resulted in larger lytic lesions in the right than in the left tibias. This *in vivo* experiment supported our hypothesis that mTOR inhibition can inhibit the paracrine activity of BC cells.

Therefore, besides the general anti-tumor activity obtained by mTOR inhibition, the beneficial bone-sparing result in the BOLERO-2 trial may be explained by at least two effects. The first mechanism includes the known direct inhibition of OC activity [17, 41], while the second acts through a negative regulation of the paracrine secretion of pro-OC factors by BC cells. An additional OC-independent effect is also related to the pro-osteoblast activity of Everolimus, as recently reported [50]. The definite effect of mTOR inhibition on bone metastatic cancers was recently explored in a collaborative review [51].

Finally, the potential of a sub-lethal dose of Everolimus to abrogate the paracrine pro-OC activity of BC cells acquires additional relevance when translated to the clinical setting. In fact, in disseminated tumor cells (DTC) PI3K/Akt signaling is reduced, thus suggesting that mTOR pathway inhibition might be linked to a biologic quiescence of DTC [52]. Thus, Everolimus at sub-lethal dose alone, or in association with other treatments, may delay or disable dormant bone metastatic disease by freezing the tumor cells living in a quiescent state in bone niches, which can generate metastases even after long periods of apparent disease remission [53].

## Conclusions

Two major points emerge from an analysis of our work. The first is the capacity of Everolimus to restrain the progression of skeletal metastatic disease in BC patients through a specific mechanism that includes inhibiting the release by these cells of the most effective pro-OC factors such as M-CSF, TNFα, IL-1β, IL-6, MIP-1α and MMP-13. The second point concerns the molecular pathway activated in the release of several of these factors. M-CSF, IL-1β and IL-6 secretion is regulated both in OCs and BC cells by the NFkB pathway, whose signaling is apparently independent of mTOR. Instead, here we provide evidence that these pathways are interconnected and that the inhibitory effects on mTOR exerted by Everolimus include a negative regulation of NFkB, thus explaining the reduced secretion of M-CSF, IL-1β and IL-6 by BC cells after mTOR inhibition. Therefore, the beneficial effects of Everolimus on bone health reported in the BOLERO-2 trial are related to the inhibition of the pro-OC paracrine activity of BC cells. To our knowledge, this is the first report describing this specific effect of the mTOR inhibitor.

## Additional file

Additional file 1: Figure S1. Measurement of Everolimus cytotoxicity on BC cell lines. **Table S1.** Primer sequences used for Real-Time PCR. **Table S2.** Optical density (OD) values, normalized to β-actin, by Western blot analyses. (DOCX 78 kb)

## Abbreviations

Akt: Protein Kinase B
BC: Breast cancer
Cat-K: Cathepsin K
c-fms: Colony-stimulating factor-1 receptor
CM: Conditioned medium
EGFR: Epidermal growth factor receptor
IC: Inhibitory concentration
IGFR: Insulin growth factor receptor
InsR: Insulin receptor
IKK: IkB kinase
IL: Interleukin
MCP-1: Monocyte chemoattractant protein-1
M-CSF: Macrophage-colony stimulating factor
MIP-1α: Macrophage inflammatory protein 1 alpha
MMP: Metalloproteinase
mTOR: Mammalian target of Rapamycin
NFkB: Nuclear factor kappa B
OC: Osteoclast
PBMCs: Peripheral blood mononuclear cells
PI3K: Phosphoinositide 3-kinase
PIK3CA: Catalytic subunit of PI3K
PTHrP: Parathormone-related protein
RANKL: Receptor activator of NFkB ligand
SCID: Severe combined immunodeficient
TNF: Tumor necrosis factor
TRAcP: Tartrate resistant acid phosphatase
