# Supplementary material for: Everolimus restrains the paracrine pro-osteoclast activity of breast cancer cells
Source: BMC Cancer. 2015 Oct 14;15:692. doi: 10.1186/s12885-015-1717-8 (PMC4606500; doi:10.1186/s12885-015-1717-8)
Supplement: Additional file 1: Figure S1. — Measurement of Everolimus cytotoxicity on BC cell lines. Table S1. Primer sequences used for Real-Time PCR. Table S2. Optical density (OD) values, normalized to β-actin, by Western blot analyses. (DOCX 78 kb) [file 12885_2015_1717_MOESM1_ESM.docx]

**Supplemental data**

**Fig. S1**

**0**

**20**

**40**

**60**

**80**

**100**

**120**


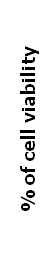


**10^-1^**

**1**

**10**

**10^2^**

**10^3^**

**10^4^**

**nM**

**Everolimus**

**0**

**MCF-7**

**MDA-MB-231**

**IC20**

**(0.5nM)**

**IC20**

**(10nM)**

**Fig. S1 - Measurement of Everolimus cytotoxicity on BC cell lines.**

Both MDA-MB-231 and MCF-7 cell lines were treated for 48 hrs with increasing concentrations of Everolimus. MCF-7 generally showed a higher sensitivity than MDA-MB-231. The IC_20_ was established as 0.5 nM for MCF-7, and 10 nM for MDA-MB-231.

| **Gene Symbol** | **GenBank accession N** | **Forward primer 5’>3’** | **Reverse primer 5’>3’** | **Amplicon size (bp)** |
| --- | --- | --- | --- | --- |
| ***M-CSF*** | NM_000757 | TGCGCTTCAGAGATAACACCCC | TCTCATAGAAAGTTCGGACGCAGG | 132 |
| ***RANKL*** | NM_033012 | GATCACAGCACATCAGAGCAG | AAGAGGACAGACTCACTTTATGGG | 153 |
| ***TNF-α*** | NM_000594 | ACTTTGGAGTGATCGGCC | GCTTGAGGGTTTGCTACAAC | 139 |
| ***IL-1β*** | NM_000576 | ACGATGCACCTGTACGATCACTG | TGTACAAAGGACATGGAGAACACC | 148 |
| ***IL-6*** | NM_000600 | ACAGCCACTCACCTCTTCAGAAC | AGCCATCTTTGGAAGGTTCAGGTTG | 157 |
| ***MMP-9*** | NM_004994 | ACTTTGACAGCGACAAGAAGTGGG | ATGCCATTCACGTCGTCCTTATGC | 184 |
| ***MMP-13*** | NM_002427 | TTGAGCTGGACTCATTGTCGGGC | TCTCGGAGCCTCTCAGTCATGGAG | 176 |
| ***MCP-1*** | NM_002982 | AATCAATGCCCCAGTCACCTGC | ACAGATCTCCTTGGCCACAATGGTC | 145 |
| ***MIP-1α*** | NM_002983 | TCTCTGCAACCAGTTCTCTGCATC | TTTCTGGACCCACTCCTCACTGG | 202 |
| ***TRAcP*** | NM_001611 | TGCATACTCTAAGATCTCCAAGCG | AGTCATCTGAGTTGCCACATAGTG | 137 |
| ***c-fms*** | NM_005211 | GAGTTCCTCTTCACACCAGTGG | AGTATAACTGTTGCCCTCATAGCTC | 153 |
| ***Cat-K*** | NM_000396 | GAAGACCCACAGGAAGCAATATAAC | TATGGACACCAAGAGAAGCCTC | 119 |

**Table S1**. Primer sequences used for Real-Time PCR

**Table S2.** Optical density (OD) values, normalized to β-actin, by Western blot analyses.

|  | **Untreated**  **MDA-MB-231** | **RAD001-treated**  **MDA-MB-231** | **Untreated**  **MCF-7** | **RAD001-treated**  **MCF-7** |
| --- | --- | --- | --- | --- |
| **Akt** | 1.78 | 1.76 | 2.19 | 1.92 |
| **p-mTOR** | 1.86 | 0.75 | 2.19 | 0.39 |
| **p-p70S6K** | 2.63 | 1.50 | 4.52 | 3.96 |
| **IKKα** | 3.59 | 2.57 | 2.08 | 2.20 |
| **p-IKKα** | 1.90 | 0.20 | 1.01 | 0.42 |
| **p65** | 2.91 | 2.69 | 2.45 | 2.54 |
| **p-p65** | 2.79 | 2.23 | 4.05 | 2.21 |
| **M-CSF** | 3.33 | 2.53 | 4.23 | 1.65 |
| **IL-6** | 2.68 | 1.68 | 4.46 | 1.39 |
| **IL-1β** | 1.76 | 0.88 | 1.79 | 0.12 |
| **MIP-1α** | 1.58 | 1.22 | 1.99 | 1.44 |
| **TNF-α** | 1.77 | 1.33 | 1.49 | 0.87 |
